# Transcatheter Left Atrial Appendage Closure

**DOI:** 10.14797/mdcvj.1215

**Published:** 2023-05-16

**Authors:** Gordon X. Wong, Gagan D. Singh

**Affiliations:** 1 University of California, Davis Medical Center, Sacramento, California, US

**Keywords:** percutaneous left atrial appendage closure, left atrial appendage occlusion, atrial fibrillation, anticoagulation intolerance, dual antiplatelet therapy, intracardiac echocardiography

## Abstract

Atrial fibrillation is the most common arrhythmia worldwide, placing a large population at risk for potentially disabling ischemic strokes, yet an estimated 50% of eligible patients cannot tolerate or are contraindicated to receive oral anticoagulation. Within the last 15 years, transcatheter options for left atrial appendage closure (LAAC) have provided a valuable alternative to chronic oral anticoagulation for reducing risk of stroke and systemic embolism in patients with nonvalvular atrial fibrillation. With newer generation devices such as Watchman FLX and Amulet gaining approval from the US Food and Drug Administration in recent years, several large clinical trials have demonstrated the safety and efficacy of transcatheter LAAC in a population intolerant to systemic anticoagulation. In this contemporary review, we discuss the indications for transcatheter LAAC and the evidence evaluating the use of various device therapies currently available or in development. We also examine current unmet challenges in intraprocedural imaging and controversies in postimplantation antithrombotic regimens. Several ongoing seminal trials are hoping to clarify the role of transcatheter LAAC as a safe, first-line option for all patients with nonvalvular atrial fibrillation.

## Introduction

Atrial fibrillation (AF) is the most common arrhythmia, affecting nearly 3% of the total population and with a prevalence of 17% in people aged 80 years and older.^1^ In the absence of systemic anticoagulation, AF leads to a 5-fold increase in the rate of ischemic stroke, a leading cause of disability in the United States (US) that leads to increased mortality and morbidity.^1^ The left atrial appendage (LAA), a vestigial remnant of the embryonic left atrium (LA), serves as the nidus for thrombus formation in patients with AF and has been well-characterized by transesophageal echocardiography (TEE).^2^ In AF, remodeling of the LAA through dilation, stretching, and reduction in pectinate muscle volume leads to local stasis, endocardial damage, and hypercoagulable activation, completing Virchow’s triad.^3^,^4^ While long-term oral anticoagulation (OAC) has been well-established as the first-line therapy to reduce the risk of AF-related stroke and systemic embolization, many patients experience increased bleeding risk and intolerance to long-term OAC, presenting a strong contraindication to their use.

Within the past 15 years, many percutaneous LAA closure (LAAC) devices have been introduced to address the challenge of stroke and systemic embolization reduction in patients with contraindications to long-term OAC. In this review, we discuss the indications and contraindications for percutaneous LAAC, current device solutions in market and development, intraprocedural imaging modalities, and postimplantation antithrombotic recommendations.

## Indications and Contraindications for Percutaneous LAAC

### Indications

Both the 2019 AF management guidelines by the American College of Cardiology and the American Heart Association and the 2016 AF guidelines by the European Society of Cardiology and the European Stroke Organization give a class IIb recommendation (Level of Evidence B-NR) to consider percutaneous LAAC in patients with increased risk of stroke and contraindications to long-term anticoagulation.^5^,^6^ The majority of institutions define a high thromboembolic risk as having a CHA_2_DS_2_-VASC score ≥ 3 or CHADS_2_ score ≥ 2. The Centers for Medicare & Medicaid Services (CMS) outlines the use of US Food and Drug Administration (FDA)-approved LAAC devices for nonvalvular AF in patients with: (1) CHADS_2_ score ≥ 2, (2) documentation of shared decision-making using an evidence-based tool on oral anticoagulation suitability with an independent noninterventional physician, (3) suitability for short-term (but not long-term) anticoagulation, (4) performed by a proceduralist with sufficient experience in LAAC device implantation, and (5) enrollment in a national, audited outcomes-based registry.^7^ Other potential indications for percutaneous LAAC described in the literature are shown in Table 1.^8^

**Table 1:** Indications for percutaneous left atrial appendage closure (LAAC). OAC: oral anticoagulation


**Primary indication for percutaneous LAAC**

Elevated thromboembolic risk (CHA_2_DS_2_-VASC > 3)

Contraindicated for long-term OAC

**Potential indications for percutaneous LAAC**

High risk of bleeding with OAC due to: Severe renal or hepatic dysfunction Inherited/acquired bleeding disorders or coagulopathies Increased risk of intracerebral hemorrhage (ie, arteriovenous malformation, brain metastases) High probability of severe or recurrent trauma (ie, elderly or patients with epilepsy)

Presence of thromboembolic phenomena while on therapeutic OAC

Chronic OAC intolerance (ie, severe gastrointestinal disease)

Employed in high-risk occupations

Unwilling to take or comply with OAC


According to the National Cardiovascular Data Registry (NCDR), increased thromboembolic risk, history of major bleeding, and high fall risk were the top three commonly reported indications for percutaneous LAAC implantation.^9^

Under current coverage recommendations set by the CMS, LAAC is “only covered as a second-line therapy” to OAC. Two large randomized controlled trials, CHAMPION-AF and CATALYST, are currently underway to evaluate the safety and efficacy profiles of the Watchman FLX (Boston Scientific) and Amulet (St. Jude Medical) devices, respectively, against OAC for protection against stroke and systemic embolization in a broad population of nonvalvular AF patients.^10^,^11^ With CHAMPION-AF and CATALYST trials in progress, the much-awaited results will be pivotal in deciding whether device-based therapy is a safe and effective first-line option.

### Contraindications to LAAC

Several patient- and procedure-related factors should be considered to assess the suitability of percutaneous LAAC procedures since they can present as potential contraindications. Based on current guidelines, patients at low thromboembolic risk with a CHA_2_DS_2_-VASC score < 3 or CHADS_2_ score < 2 are not recommended for LAAC. In addition, patients who cannot tolerate short-term OAC or dual antiplatelet therapy (DAPT) are also contraindicated to undergo percutaneous LAAC.^7^,^8^ Other indications for long-term or lifelong OAC, such as mechanical heart valves, deep vein thrombosis or pulmonary embolism, or atrial/ventricular thrombi, are also contraindications for percutaneous LAAC.^9^

Patients with AF related to valvular heart disease such as mitral stenosis were excluded from PROTECT-AF and PREVAIL trials evaluating Watchman against OAC, and without large registry or clinical trial data, patients with valvular AF are currently not indicated for percutaneous LAAC.^9^,^12^ Factors such as the presence of left atrial thrombus or tumor, active infection, or prior atrial septal defect/patent foramen ovale closure devices limit the use of transseptal puncture, thereby making percutaneous LAAC technically challenging.^9^,^13^ However, with proper CT planning, LAAC can still be performed successfully in patients with prior septal occlusion devices.^14^ Finally, suitable LAA anatomy with comparable appendage depth and width is important for safe device implantation, although this is less of a concern with current generation devices.

TEE is commonly used for device deployment, so patients who cannot tolerate TEE are not ideal candidates to receive percutaneous LAAC. Esophageal conditions (stricture, varices, inflammation, perforation), history of upper gastrointestinal bleeding, and restricted neck mobility are common relative contraindications that can limit the use of TEE. While intracardiac echocardiography (ICE) is an emerging alternative, TEE remains the gold standard and the most widely available imaging modality. Imaging by ICE versus TEE is discussed later in this review.

## Percutaneous Laac Device Therapies

### Watchman

**Figure 1:**
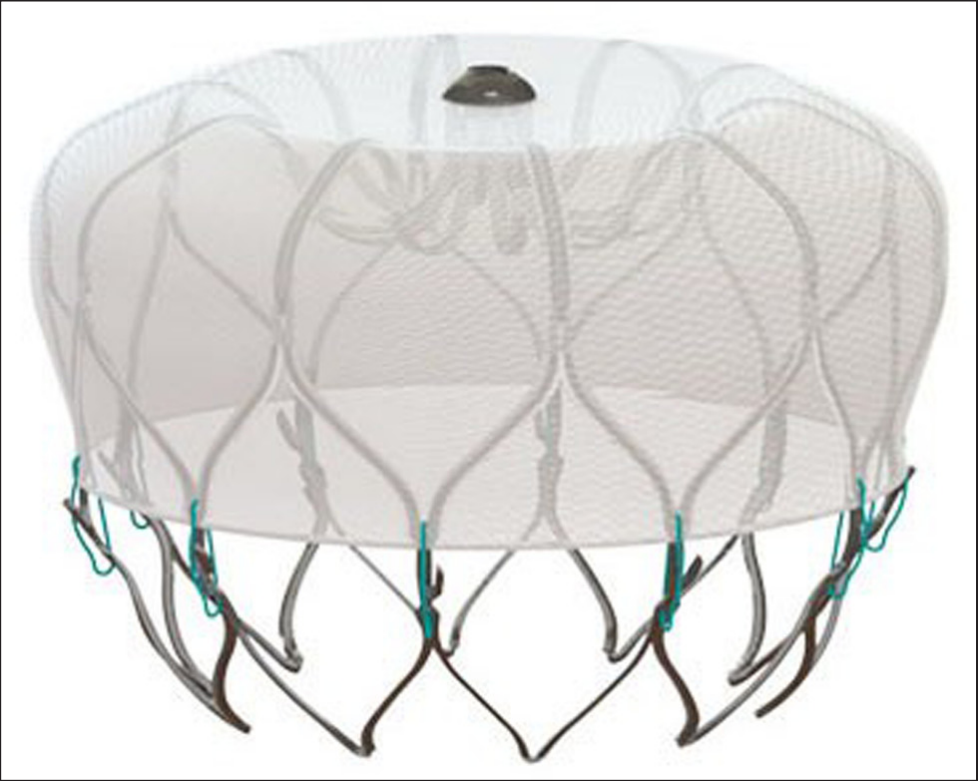
Watchman. Reproduced with permission from Boston Scientific. ©*2023 Boston Scientific Corporation or its affiliates. All rights reserved*.

Although no longer available, the original FDA-approved Watchman device (Figure 1), sometimes referred to as Watchman 2.5, was a self-expanding occlusion device designed to close off the body of the LAA. Gaining FDA approval on March 13, 2015, it consisted of a 10-strut spherical nitinol frame, with a 160 μm polyethylene terephthalate fabric mesh cap on the exterior designed as a filter to prevent harmful embolization from the healing process and promote device endothelialization. The polyethylene terephthalate cap faced the body of the LA while the open distal end contained 10 active fixation anchors to the LAA. The device is placed using transseptal catheter-based approach using fluoroscopy and TEE guidance. Available in five sizes ranging from 21 mm to 33 mm, Watchman was used to occlude LAA diameters of 17 mm to 31 mm, with proper device sizing being 8% to 20% larger than the LAA diameter to allow for adequate compression. In addition, sufficient LAA depth was needed to accommodate the device, which was equal in length and diameter when fully expanded. The device could be partially recapturable and repositioned if deployed too distally into the LAA and fully recaptured for removal if placed too proximally.

PROTECT-AF was a multicenter randomized controlled trial of 707 patients with nonvalvular AF and CHADS_2_ score ≥ 1 randomized in a two-to-one fashion to either LAA closure with Watchman or the warfarin control group. Patients in the Watchman arm were maintained on warfarin for 45 days after implantation, followed by DAPT with aspirin and clopidogrel for 6 months postprocedure and then aspirin monotherapy indefinitely. The trial results showed LAA closure with Watchman as non-inferior to warfarin therapy in preventing cardiovascular death, stroke, and systemic embolism in nonvalvular AF patients. However, more periprocedural complications such as serious pericardial effusions were seen in the Watchman arm, while more hemorrhagic strokes were seen in the warfarin control group.^15^

The PREVAIL trial was conducted specifically to further evaluate the safety end point of the Watchman device and mandated the inclusion of at least 25% of new operators to further assess the effect of procedural performance. Randomizing an additional 407 patients in a two-to-one fashion to Watchman or warfarin, the trial results failed to demonstrate non-inferiority in the composite of stroke, systemic embolism, and cardiovascular death, which was attributed to a lack of statistical power and low rates of ischemic stroke in the warfarin control arm due to small sample size. The trial did show better implant success rates, with 93% success in new operators, and less procedure-related complications. Overall, non-inferiority was established between Watchman and warfarin in preventing stroke and systemic embolism.^16^

### Watchman FLX

**Figure 2:**
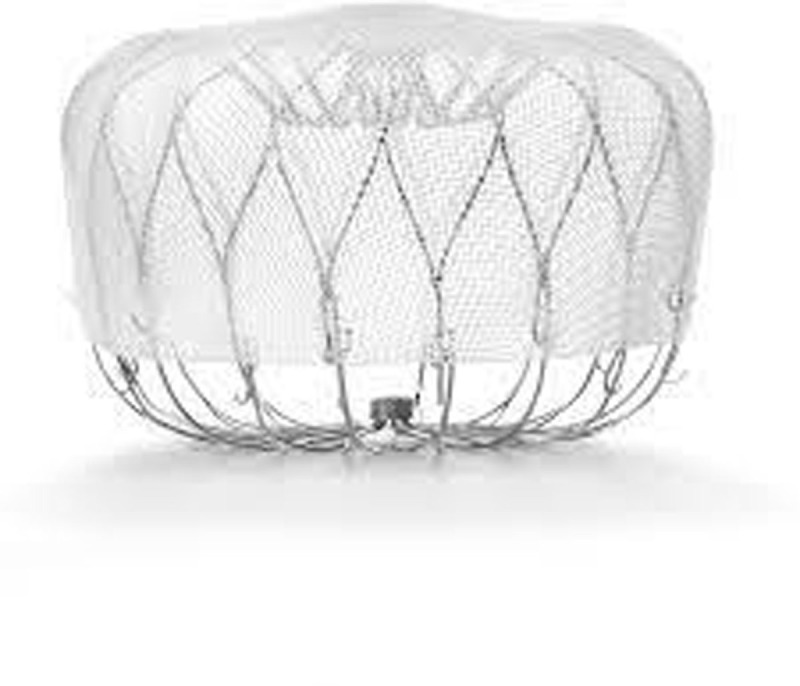
Watchman FLX. Reproduced with permission from Boston Scientific. ©*2023 Boston Scientific Corporation or its affiliates. All rights reserved*.

The Watchman FLX (Figure 2) is the current-generation device aimed to accommodate a wider range of LAA morphologies, coming in five device sizes ranging from 20 mm to 35 mm to treat LAA ostia diameters of 15 mm to 32 mm. It is 10% to 20% shorter in length compared to the previous generation model and contains an atraumatic closed distal end to reduce the risk of perforation, pericardial effusion, and cardiac tamponade. Other redesign aspects include increasing the number of struts to 18 for improved tissue fixation and radial strength, and adding dual row anchors to reduce device embolization risk. The device is fully recapturable and repositionable. It received Conformité Européenne (CE) marked approval in March 2019 and gained FDA approval in July 2020 following results from the PINNACLE FLX trial.

PINNACLE FLX was a prospective nonrandomized trial enrolling 400 nonvalvular AF patients to receive LAAC with Watchman FLX. Patients received direct oral anticoagulants with aspirin for 45 days postimplantation and were transitioned to DAPT for 6 months postprocedure if no peridevice leak (PDL) > 5 mm was seen on TEE at 45 days. Afterwards, they were maintained on aspirin monotherapy. There was a 98.9% implant success rate, and the trial met its primary safety and efficacy end point, demonstrating a low rate (0.5%) of major procedure-related safety events at seven days postprocedure and 100% LAA closure with < 5 mm PDL at 12 months postprocedure. While no serious pericardial effusions were seen within 7 days of implantation, three cases required percutaneous drainage between 7 and 45 days. Device-related thrombus (DRT) occurred in seven patients at 12 months follow-up, with embolic events in two patients and no early or late device embolization.^17^ Implant success rates and low adverse event rates continue to be favorable on postapproval registry publications.^18^

### Amulet

**Figure 3:**
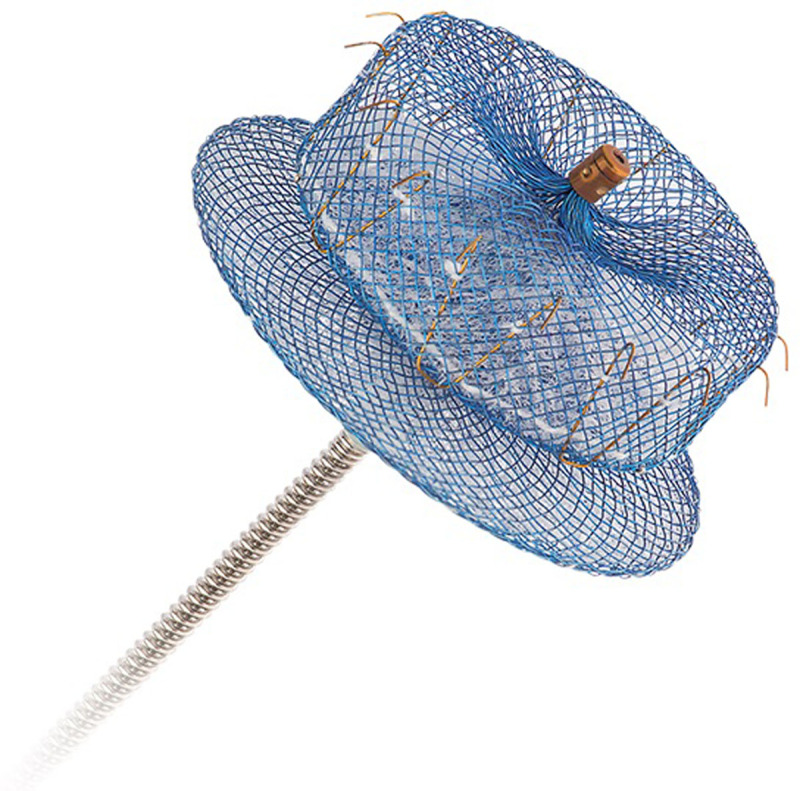
Amulet cardiac plug. Reproduced with permission from Abbott Vascular.

The Amulet (Abbott Vascular) is the latest generation of the Amplatzer Cardiac Plug, a self-expanding device of nitinol mesh with polyester fabric (Figure 3). Shorter in length than in diameter, the Amulet is designed to fit shorter LAA anatomies and is available in 8 sizes ranging from 16 mm to 34 mm diameter. Conformable and able to accommodate a variety of LAA morphologies, the device’s lobe component is positioned within the LAA neck, using stabilizing wires to anchor within the LAA body. The disc, connected to the lobe by a flexible but tension-providing waist, seals the LAA orifice, excluding the LAA from the LA.

The Amulet gained CE approval in 2013 and FDA approval on August 14, 2021, following results from the Amplatzer Amulet Left Atrial Appendage Occluder trial, known as Amulet IDE. This was a prospective trial evaluating the safety and efficacy of Amulet, randomizing 1,878 patients with nonvalvular AF to LAAC with either Amulet or Watchman (control group). Device implantation was guided by TEE and fluoroscopy, with high success rates in both groups. In the Amulet group, 76% of patients were discharged on DAPT (aspirin and clopidogrel) while 20% received aspirin with OAC, and in the Watchman group, 82% of patients received aspirin with warfarin at discharge. For the primary safety end point, Amulet demonstrated non-inferiority with Watchman in the composite of procedure-related complications, all-cause death, or major bleeding through 12 months. Procedure-related complications were higher in the Amulet group due to more frequent pericardial effusions and device embolization, which occurred earlier in the Amulet implanter experience. For the primary efficacy end point, Amulet was non-inferior to Watchman in the rate of ischemic stroke and systemic embolism at 18 months. Amulet demonstrated superiority over Watchman in terms of successful device-based LAA occlusion, defined as residual jet less than 5 mm, which was observed in 98.9% of Amulet patients compared to 96.8% of Watchman patients. Moreover, complete occlusion with no peridevice residual jet was found in 63% of Amulet patients and 46.1% of Watchman patients. Low rates of DRT at 18 months were seen in both groups, although two Watchman patients with DRTs experienced thromboembolic events.^19^

### LARIAT

The LARIAT (SentreHEART Inc) is a percutaneous LAA suture that utilizes snare-ligation to exclude the LAA. While used off-label for LAA exclusion, it originally gained FDA 510k clearance in 2006 as a pre-tied suture for soft tissue approximation during surgery. Using transseptal and pericardial access, the LARIAT device is delivered via a pericardial sheath over a rail system created by epicardial and endocardial guidewires.^20^ A pericardial drain is typically left in place for 24 to 48 hours afterwards and patients are discharged on antiplatelet therapy, with nonsteroidal anti-inflammatory drugs and short-term colchicine for pain relief.

Multiple single-center trials evaluating the efficacy and safety of LARIAT have seen procedural success balanced by a high rate of procedural complications, such as pericarditis, pericardial effusion (requiring pericardiocentesis), major bleeding, and pleural effusions.^20^ In July 2015, the FDA published a safety communications warning regarding off-label use of LARIAT for LAAC weeks after granting investigational device exemption approval for device inclusion in a randomized controlled trial comparing LAA ligation with pulmonary vein isolation (PVI) against PVI alone in AF patients. The trial did not find superiority in adding LAA ligation to PVI in preventing recurrent AF.^21^

### LAmbre

The LAmbre (Lifetech Scientific) is a self-expanding occlusion device composed of a 10.4F to 12.3F delivery sheath, distal umbrella, and proximal cover disc. The distal umbrella, comprised of nitinol mesh and polyester membrane, contains eight small hooks to engage LAA walls and eight U-shaped ends to stabilize against muscular trabeculations. The proximal disc cover, made of polyethylene terephthalate, orients to the LAA wall. Fully retrievable and repositionable, the device earned CE approval in 2016.

A systematic review of 10 clinical trials from 2016 to 2019 with 403 nonvalvular AF patients implanted with LAmbre showed a 99.7% implantation success rate and a 2.9% composite procedural complication rate, including death, stroke, pericardial tamponade, and major bleeding.^17^ No device embolization or major bleeding events were reported. Over 96% of patients received DAPT for 3 to 6 months postimplantation, with a 1.7% rate of stroke/TIA and 0.7% rate of DRT up to 12 months.^22^ The low DRT rate was attributed to LAmbre’s proximal disc cover closing the LAA ostium and reducing PDL-associated thrombogenicity.^22^ While several trials excluded patients with left ventricular ejection fraction ≤ 40%, future studies on LAmbre’s efficacy and safety in this population are needed.

Several small trials comparing LAmbre with Watchman and Amulet have suggested comparable efficacy and safety profiles, although trials with larger enrollments are needed.^23^,^24^,^25^ LifeTech gained FDA approval in March 2022 for a pre-market randomized multicenter trial comparing the safety and efficacy of LAmbre against OAC. The trial is anticipated to enroll more than 3,000 patients, with more than 1,500 implantations.

### Conformal

**Figure 4:**
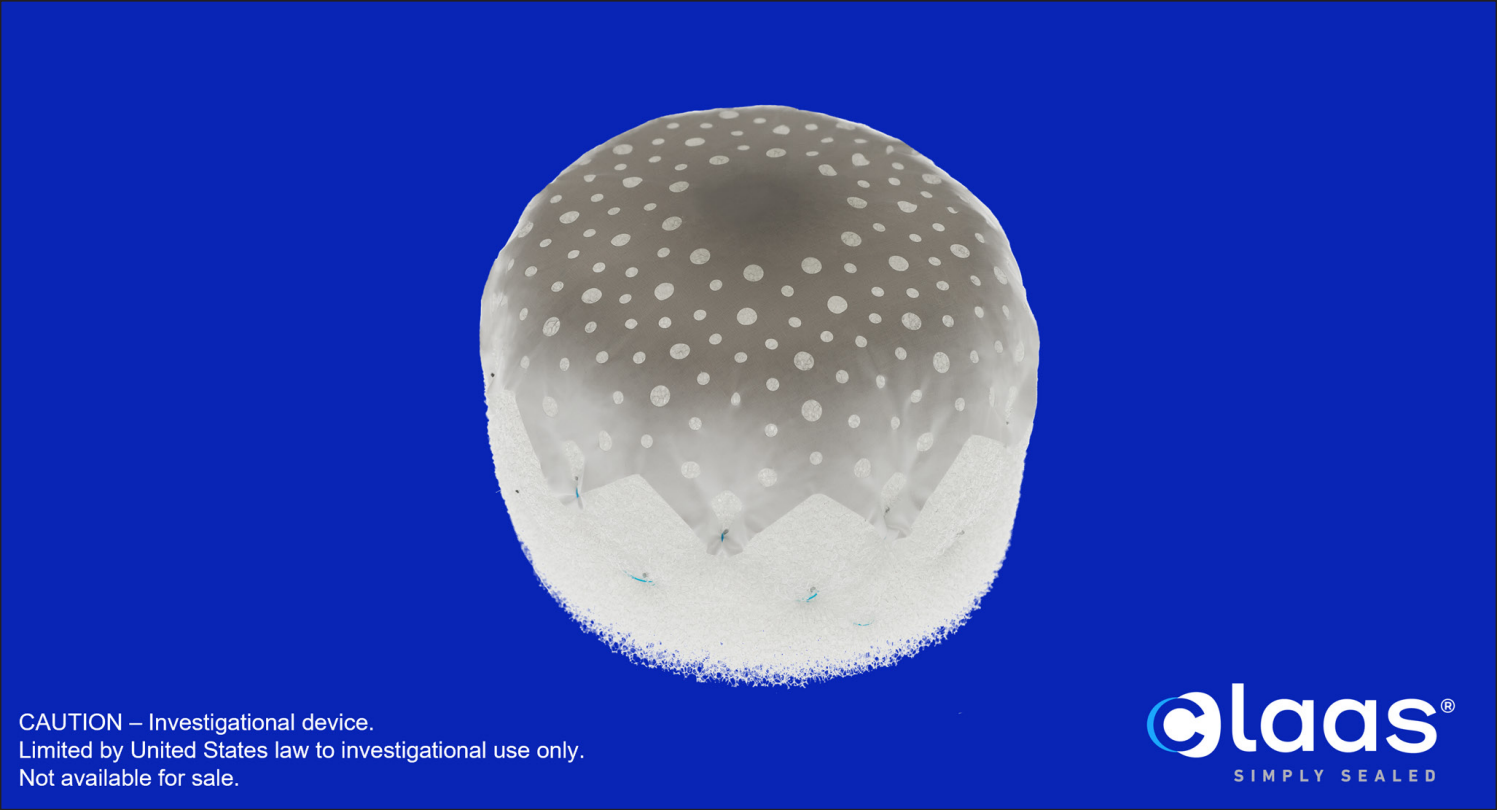
Conformal left atrial appendage seal. Reproduced with permission from Conformal Medical.

The Conformal LAA Seal (Conformal Medical) is a novel self-expanding device composed of nitinol and covered by a porous polyurethane-carbonate matrix foam serving as an atraumatic distal end (Figure 4). The proximal (left atrial) face is a polytetrafluoroethylene cover that allows for recapture and minimizes thrombogenicity. Compression of the device in one dimension results in expansion in a perpendicular dimension, allowing the device to accommodate various LAA anatomies. Two sizes (27 mm and 35 mm) exist to cover LAA ostia diameters ≤ 25 mm and ≤ 32 mm, respectively, and require a landing zone of 10 mm. The delivery system is introduced via transseptal access, and the implant can be recaptured and redeployed.

The first prospective clinical study evaluated the efficacy and safety of Conformal in 15 nonvalvular AF patients. Following preprocedural TEE for echocardiographic eligibility and device sizing, successful ICE-guided implantation was achieved in all patients, although four patients required a second or third device. Patients received 6 months of DAPT followed by single antiplatelet therapy. At 45-day follow-up, two small residual leaks of 1 mm to 2 mm were seen on TEE, and at 6- and 12-month follow-up, only one patient had a 1 mm leak. There were no stroke/TIA, device embolization, major bleeding, or vascular injury events. One patient had a moderate-sized DRT at 6 months that was successfully treated with subcutaneous low-molecular weight heparin, and the two deaths at 12-month follow-up were unrelated to the procedure or device implantation.^26^,^27^

A second study of 22 patients undergoing TEE-guided Conformal implantation under general anesthesia reported 82% implantation success rate; however, the implant was recaptured in four patients due to inadequate seal. All 18 patients received 6 months of DAPT followed by aspirin monotherapy. Four patients experienced major adverse events at 45-day follow-up, with one death from bilateral pulmonary emboli, two patients with significant bleeding requiring blood transfusion, and one patient with DRT visualized by TEE, which resolved with 6 months of warfarin therapy without any thromboembolic sequelae. Throughout the study period, one patient had a persistent significant leak due to the presence of an unrecognized large posterior lobe at the time of implantation. At 12 months, there were no reports of device embolization, DRT, stroke/TIA, or new residual leaks in the remaining patients.^28^

## Intraprocedural Imaging

TEE remains the conventional standard for intraprocedural imaging in LAAC implantation cases, providing high quality imaging for procedural success. However, it is invasive, necessitating general anesthesia, and requires a dedicated TEE operator, which increases procedural costs and lengthens recovery time. TEE used in structural heart interventions has been associated with esophageal or gastric injury, with longer procedural times and poor/suboptimal image quality as risk factors.^29^ Recently, there has been increasing interest in the use of ICE as the sole means to guide LAAC. Utilizing contralateral transvenous access, ICE can help guide transseptal access, after which the catheter can be advanced directly into the LAA either via the same or different transseptal puncture.^30^ From there, the ICE catheter can provide suitable imaging to help guide the procedure and provide the necessary components that meet manufacturer specified device deployment criteria.

**Figure 5:**
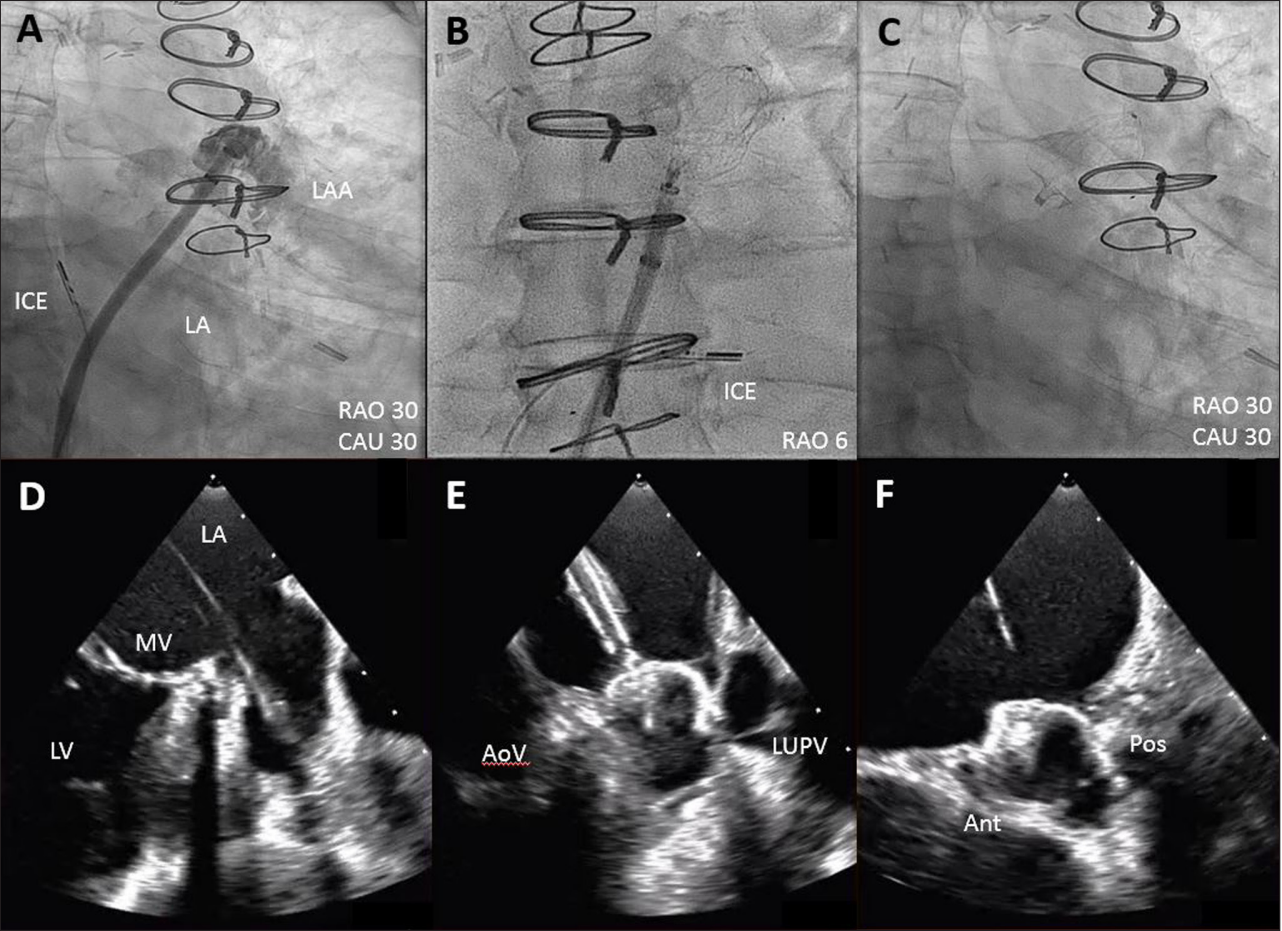
ICE-guided left atrial appendage closure. Fluoroscopic images of the ICE-guided LAAC with Watchman FLX: **(A)** Mid left atrial position of the ICE catheter during left atrial appendogram. **(B)** Mitral valve inflow position of ICE catheter following device implantation. **(C)** Successful deployment of 31-mm Watchman FLX. **(D-F)** Simplified ICE imaging of the LAAC procedure. Ant: anterior; AoV: aortic valve; CAU: caudal; LA: left atrium; LUPV: left upper pulmonary vein; LV: left ventricle; Pos: posterior; RAO: right anterior oblique

ICE-guided LAAC (Figure 5) can be performed with conscious sedation, which may improve patient safety and satisfaction by eliminating the time and risks associated with intubation and extubation.^31^ Several cost-effectiveness analyses comparing ICE and TEE for LAAC procedures have shown improved efficiency in catheterization lab logistics from ICE use, with shorter procedural, in-room times, and lab turnaround times.^30^,^32^,^33^,^34^,^35^ However, these studies found no significant difference in terms of total hospital charges comparing ICE and TEE use, where the high cost of single-use ICE catheters was often balanced by the professional fees and recovery room charges related to TEE use. Due to shorter recovery periods, same-day discharges after ICE-guided LAAC have been reported, showing a 15% reduction in total hospital costs and without further adverse events.^36^ Finally, ICE-guided LAAC may mandate the use of CT for pre-procedure planning to help implanters understand the anatomy of the LAA being treated.

## Post-implantation Antithrombotic Regimens

Antithrombotic regimens are maintained following device implantation to decrease the risk of DRT before device endothelialization, which is associated with a 4-fold risk of thromboembolism and stroke.^37^ Despite the success and progress of current LAAC devices, inadequate device closure of the LAA ostium, due to limitations of current device designs and the heterogeneous anatomy of the LAA, remains an unmet clinical challenge. As residual PDL and associated DRT can lead to potentially life and limb-threatening thromboembolic consequences, the development of new devices has aimed to address this area of need.^18^,^26^

However, a uniform antithrombotic regimen following LAAC implantation has yet to be established by guidelines. Registry data has highlighted the variability of antithrombotic regimens following LAAC, in part due to novel OACs not achieving FDA approval at the time of PROTECT-AF or PREVAIL’s trial design.^38^ The NCDR Left Atrial Appendage Occlusion (LAAO) registry data on 31,944 Watchman implantations from January 1, 2016, through November 31, 2018, show that only 36.9% of patients were maintained on aspirin and warfarin after device implantation, with novel OAC usage seen in 33.1% of patients.^38^ In addition, the European Registry on Watchman Outcomes in Real-Life Utilizations shows DAPT usage in 60% of cases, vitamin K antagonist in 16%, novel OAC in 11%, single antiplatelet in 7%, and no antithrombotic regimen in 6%.^39^ Since 2017, European labeling has included the use of either postimplantation OAC or DAPT in Watchman devices, with the US following suit in 2022.

Regarding PROTECT-AF and PREVAIL trial protocol, the antithrombotic regimen after Watchman implantation consists of aspirin 81 mg to 325 mg indefinitely and warfarin for 45 days. If TEE at 45 days rules out DRT or PDL, warfarin is substituted with clopidogrel 75 mg for 6 months postprocedure.^15^,^16^ However, recent evidence from newer-generation devices has shifted away from OAC. From the Amulet IDE trial, DAPT use was approved for Amulet in August 2021, with OAC recommended only when PDL is greater than 5 mm. In September 2022, an FDA-approved label change permitted DAPT as an alternative to OAC plus aspirin in the 45-day postimplantation period for Watchman FLX, based on NCDR LAAO registry data.^8^ Recent Watchman FLX trial results presented at the Transcatheter Valve Therapy 2022 summit show no differences in the rate of DRT or the composite of death, stroke, or major bleeding comparing DAPT with DOAC or warfarin-based regimens following device implantation.^40^ The results of the ANDES trial (Short-Term Anticoagulation Versus Antiplatelet Therapy for Preventing Device Thrombosis Following Left Atrial Appendage Closure, NCT03568890) and SAFE-LAAC trial (Optimal Antiplatelet Therapy Following Left Atrial Appendage Closure, NCT03445949) will help clarify the role and duration of DAPT after LAAC.^41^,^42^

## Conclusion

Transcatheter LAAC is an effective and safe option to reduce the risk of stroke and thromboembolic events in patients with nonvalvular AF who cannot tolerate long-term OAC. Proper patient selection must consider all indications and contraindications for transcatheter closure. While Watchman and Amulet are FDA-approved for commercial use, other technologies are in clinical development to address current device limitations and other areas of unmet need. Further trials and registry data are needed to evaluate the safety and efficacy of new device solutions, identify appropriate indications for use, and tailor best postimplantation protocols.

## Key Points

- The most recent American Heart Association/American College of Cardiology and European Society of Cardiology guidelines on atrial fibrillation (AF) management give a class IIb recommendation for patients with nonvalvular AF at increased stroke risk (commonly with CHADS_2_ score ≥ 2) and with contraindications to long-term oral anticoagulation to undergo transcatheter left atrial appendage (LAAC) closure. In addition, Centers for Medicare & Medicaid Services guidelines require shared decision-making involving an independent noninterventional physician and patient suitability for short-term anticoagulation.
- Patients with valvular AF, complicated structural heart disease, intolerance to any anticoagulation, or with other indications for lifelong anticoagulation are contraindicated to receive transcatheter LAAC.
- Currently, the Watchman and Amulet are the only two closure devices approved by the US Food and Drug administration (FDA) for percutaneous LAAC, and the results of CHAMPION-AF and CATALYST trials will clarify the role of transcatheter LAAC as a first-line option to reduce stroke risk in patients with nonvalvular AF.
- While TEE is the current “gold standard” for intraprocedural imaging, intracardiac echocardiography-guided LAAC is garnering interest.
- Following percutaneous LAAC implantation, a regimen of oral anticoagulation with or without aspirin for 45 days is commonly used to reduce risk of device-related thrombus. However, based on clinical trial and registry data, the FDA has recently approved dual antiplatelet therapy for 45 days as an alternative in patients receiving both Watchman FLX and Amulet.
